# Effects of a Diabetes Self-Management Education Program on Glucose Levels and Self-Care in Type 1 Diabetes: A Pilot Randomized Controlled Trial

**DOI:** 10.3390/ijerph192316364

**Published:** 2022-12-06

**Authors:** Rocío Romero-Castillo, Manuel Pabón-Carrasco, Nerea Jiménez-Picón, José Antonio Ponce-Blandón

**Affiliations:** 1 Centro Universitario de Enfermería de Cruz Roja, Universidad de Sevilla, Avenida de la Cruz Roja, No. 1, 41009 Seville, Spain; 2 Departamento de Enfermería de la Universidad de Sevilla, Calle Avenzoar, No. 6, 41009 Seville, Spain

**Keywords:** type 1 diabetes, health education, nurses, self-management, self-care, glycemic control

## Abstract

(1) Background: Several factors have been associated with the success of health education programs, such contact time, with better results being obtained from more intensive programs and early outcome measurement. Nurses play an essential role in educating patients with diabetes both in disease-management, therapeutic education, and healthy lifestyles promotion as well as emotion management. The objective was to evaluate the effectiveness of a nurse-led educational program based on patients with type 1 diabetes; (2) Methods: An experimental, two-group comparison design, 69 patients participated in the intervention group and 62 in control group. The control group received routine health education and follow-up. The intervention group received intensive educational program led by nurses. The effects were evaluated after 1 and 3 months of intervention; (3) Results: The differences between groups in sensor usage, knowledge, and diabetes self-care three months after the educational program were significant; (4) Conclusions: The program could help type 1 diabetes patients to improve the control rates for blood glucose. The continuous glucose monitoring sensor allowed knowing which parameters improved one and three months after the intervention. The hypothesis of the influence of the emotional state on glucose levels was confirmed.

## 1. Introduction

Type 1 diabetes (T1DM) is one of the most common chronic illnesses worldwide and it constitutes a higher risk of mental health issues, including diabetes distress, depression, anxiety, and disordered eating [1]. The National Standards for Diabetes Self-Management Education and Support encourage healthcare professionals to recognize and address the emotional burden of living with and managing diabetes [2]. The Diabetes Care and Education Specialist is an “expert and teacher who provides collaborative, comprehensive, and person-centered care and education for people with diabetes” [3,4].

Blood glucose control requires not only drug treatment but also intensive health education [5]. Diabetes self-management education and support (DSMES) is a fundamental element of care for people with diabetes. DSMES consists of providing knowledge, skills, and self-confidence to accept responsibility for their self-care [6]. Some studies have demonstrated the benefits of DSMES, including improved clinical outcomes and quality of life, with reduced hospitalizations and healthcare costs [7,8,9]. Nurses play an essential role in educating patients with diabetes both in disease-management, therapeutic education, and healthy lifestyles promotion as well as on emotion management [10,11]. All of this should be considered when designing and implementing a therapeutic education program for patients with T1DM.

Diabetes education has advanced and grown in recent years as technology has been incorporated into health care through virtual, *tele-health*, telephonic, and web mobile phone-based applications [5,12,13]. Recent research in therapeutic education includes a hands-on approach to problem solving, collaborative care, including family support and addressing psychosocial issues, behavior change, and strategies for maintaining self-management and device use [14]. With respect to the latter, technological innovation has allowed the development of continuous glucose monitoring sensors to improve glycemic control [15,16].

Recent evidence suggests some failures in the knowledge and skills of people with diabetes in the treatment administration [17]. Nurses need to provide an effective and repetitive training concerning the use of insulin treatment [5]. Primary health care is the ideal setting to address errors in treatment administration and management, teaching healthy lifestyles, and complications prevention. Several factors have been associated with the success of health education programs, such contact time, with better results being obtained from more intensive programs and early outcome measurement at the end of the intervention [18]. Better results are obtained with an educational itinerary that is structured and patient-centered [19,20].

A systematic review and meta-analysis of randomized health interventions for diabetes identified that the most common intervention types were multicomponent, clinic-based interventions of diabetes education or support alone [21]. Multicomponent, clinic-based interventions (pharmacological treatment, diet, and physical exercise) were modestly effective in improving glycemic control, with a moderate certainty of evidence [22]. In high-income countries, a dose-dependent relationship was observed between contact intensity and glycemic effectiveness, namely, more intensive educational interventions with daily sessions reported better results in glycemic control [21,23].

A systematic review about the efficacy of diabetes education in Primary Care concluded that most of the programs were conducted in group as opposed to individual interventions. Group education was focused on the reduction in hemoglobin A1c(HbA1c), lipid profile, weight loss, or improvement of diet. Education groups were always small, with an average of 10 patients. The educational content of programs included risk factors, self-monitoring of blood glucose, physical exercise, diet, adherence to treatment, stress management, and understanding of the disease [24].

Psychological burdens such anxiety, depressive symptoms, and diabetes distress is highly prevalent in patients with T1DM [11]. Compared with anxiety and depressive symptoms, diabetes distress has not been recognized adequately by the healthcare professionals [25]. The prevalence of diabetes distress is around 50% of diabetic patients and it tends to be chronic and has been significantly associated with poor glycemic control and deficiency self-care according to different studies [26,27,28]. Reducing Distress and Enhancing Effective Management for T1DM Adults (T1-REDEEM) was a randomized control trial for adults with elevated diabetes distress and poor self-care designed to compare the effectiveness of an intense education/behavior change intervention with an emotion regulation skills intervention. The education/behavior intervention consisted of one-hour online sessions that covered tips on carbohydrate counting, management of T1DM, continuous glucose monitoring, resolving hypoglycemia, and travel advice. On the other hand, the emotion regulation intervention was based on program of empowerment-based communication and motivational interviewing. After the interventions, they observed reductions in diabetes distress and significant reductions in HbA1c between baseline and 3 months (*p* = 0.003), and there were no differences between the intervention groups [27].

Continuous glucose monitoring through the use of a sensor is an established method for improving glucose levels and reducing the risk of hypoglycemia in T1DM [29]. Moreover, the addition of diabetes education has the potential to improve the outcomes of this tool. This study proposes a nurse education program for patients with T1DM in an Endocrinology Day Hospital specializing in diabetes. The study hypotheses were the following:

- The control rates for blood glucose measured by the continuous glucose monitoring sensor would be higher after the educational program compared to routine-intervention levels.
- Knowledge, emotional state, and diabetes self-care activities would improve after 1 and 3 months of a nurse educational intervention compared to the control group.
- Emotional regulation, knowledge, and diabetes self-care activities would influence blood glucose control.

## 2. Material and Methods

### 2.1. Design

This study was an experimental design, and the CONSORT checklist was followed (Supplementary Table S1). The trial protocol was previously published in ClinicalTrials.gov (ID: NCT05159843). Three hundred twenty-three outpatients with T1DM were part of the unit. There were two groups in this randomized controlled trial (intervention and control). Patients included in the intervention group participated in sessions of therapeutic education in the management and self-care of diabetes, while the subjects included in the control group had access to the standard care provided by the Endocrinology and Nutrition Unit of the hospital. The study data collection was made between January to June of the year 2022. Measurements were realized at the baseline and 1 and 3 months from the educative intervention.

### 2.2. Participants

The ethical principles for medical research on human beings set out in the latest revision of the Declaration of Helsinki were applied throughout the data collection process and the anonymity of subjects was guaranteed [30]. Written informed consent was obtained from the patients in the intervention and control groups before the study. This study received approval from the Ethics Committee of the Virgen Macarena and Virgen del Rocío Hospitals (CI. 2231-N-21).

Participants were required to meet the following criteria. Inclusion criteria: (1) Patients who met the diagnostic criteria of T1DM published by the American Diabetes Association in 2021 [28]; (2) Adults over the age of 18 years; (3) Patients able to speak, read, and listen to Spanish; (4) Patients participating in this study volunteered with signed informed consent.

Exclusion criteria: (1) Patients with cognitive impairment, (2) Patients with terminal illness or any serious brain injury; (3) Patients with reading and hearing difficulties. (4) Patients taking drugs that may affect blood glucose during basic insulin treatment, such as glucocorticoids or weight-loss drugs.

Two researchers recruited endocrine patients from a Diabetes Day Hospital during consultations and informed patients of the content and purpose of the project. A principal investigator nurse was responsible for coordinating the study and collecting the data. A diabetes nurse educator delivered the diabetes management education program. The research nurse explained the study to each patient and requested informed consent, which could be revoked by the patient at any time during the study.

**Figure 1:**
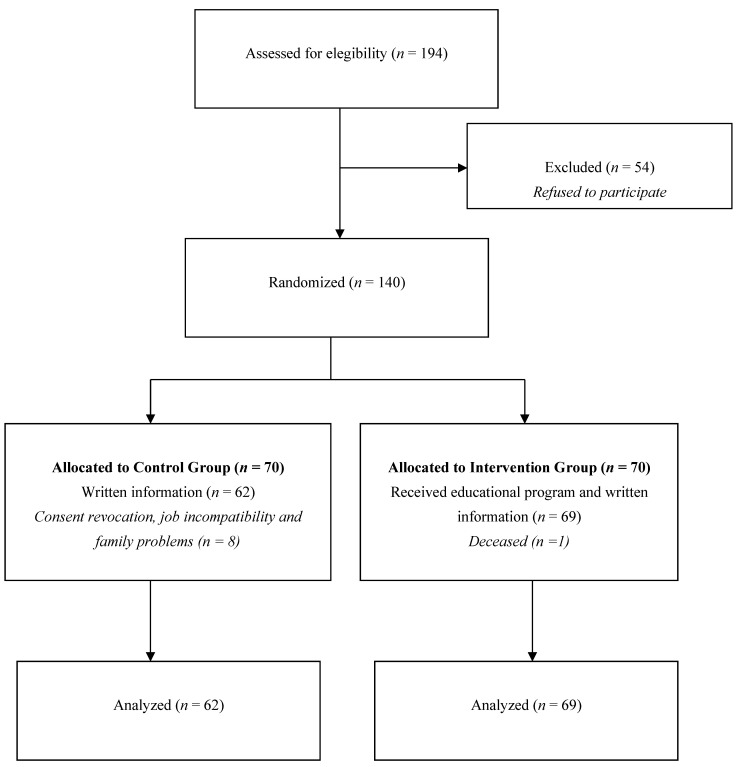
The sample flow chart and the number of participants.

They were randomly assigned to the control and intervention groups. Finally, 62 cases in the control group and 69 cases in the intervention group completed the study (Figure 1).

### 2.3. Randomization

A random number in blocks of four was generated from a computer by a researcher. Then, a sealed envelope was assigned to each patient to ensure the allocation concealment. The random allocation (1:1) of patients to each group was conducted by the principal investigator. Each patient received a serial number when they completed signing the informed consent. After finishing the baseline data collection for the four participants in each block, the research nurse opened the envelopes and determined to which groups the patients belonged. The investigator who assessed the results was blinded.

### 2.4. Routine Intervention

The diabetics in the control group received routine health education and follow-up. They received regular visits with a doctor specializing in Endocrinology and a nurse educator and standard Spanish Diabetes Society information pamphlets. Consultation care is centralized in the pharmacological treatment regimen, dosage, and guidelines. Metabolic control parameter examinations were performed during the visits and patients received routine education of individual-based counseling by physicians during each outpatient visit (15 min). The education content mainly included general knowledge on diabetes, blood glucose monitoring, and regular examinations. Patients needed to visit the doctor for a recheck at 1 and 3 months according to their diabetic control and the adequacy of treatment. Those assigned to the control group would receive the educational intervention after finishing the study, since they had the right to benefit from this education. It was something requested even by the Ethics Committee.

### 2.5. Nurse-Led Health Education

Participants randomized to the intervention group received a structured program of therapeutic education that was organized in four consecutive days for groups of four patients. The education was provided by an advanced practice nurse specializing in diabetes and she had undergone standardized training procedures. The sessions lasted for one or two hours a day with clear and concise information to guarantee the attention and concentration of the participants. Each group session was structured into four procedures: (a) theoretical explanation of the module; (b) practical exercise for participants; (c) discuss experience and resolve doubts and concerns; (d) conclusions and summary of basic points. The session’s contents are detailed in Table 1.

### 2.6. Outcome Measures and Data Collection

Data collection was made at three different time points throughout the study: baseline, 1 month, and 3 months. Sociodemographic data, such gender, age, marital status, education level, and employment status were collected at the baseline visit. The following parameters and scales were collected at three points (baseline, 1 month, and 3 months).

Glucose levels were evaluated with the continuous glucose monitoring sensor. The parameters collected by the sensor are: high range (>180 mg/dL), target range (70–180 mg/dL), low range (<70 mg/dL), average glucose (mg/dL), glycosylated hemoglobin (HbA1C, %), and sensor usage (%). The last parameter is an estimation of the time of sensor use. A brief knowledge test prepared by unit experts was performed to the patient. There were ten questions with a maximum score of ten, with one point for each question. Higher scores indicated a better mastery of diabetes knowledge.

Diabetes self-management was measured using The Summary of Diabetes Self-Care Activities (SDSCA). An adapted and validated version in Spanish was used in this study, which assessed the aspects of diet, physical activity, and self-monitoring of blood sugar. This questionnaire consists of seven items, and each item scored from zero to seven [31]. The total scores indicated better diabetes self-management behaviors.

Finally, the emotional state was assessed with the Goldberg Anxiety and Depression Scale [32]. This scale is composed of two subscales of nine binaries (yes/no) items. The cut-off point for the anxiety subscale is four or more points and two or more points for the depression subscale, higher scores indicating greater anxiety and more depressive symptoms in the patient.

The research protocol was followed, inclusion and exclusion criteria were respected, as well as randomization and allocation concealment. The follow-ups for the patients of the two groups were arranged in different time points to avoid the contamination.

The Statistical Package for Social Sciences (SPSS 22.0; IMB Corporation, Armornk, New York, NY, USA) was used for tabulation and data analysis. First, a descriptive analysis of the whole sample was made, followed by a bivariate analysis for non-parametric samples, with Spearman’s rho statistical test, because the sample was not a normal distribution. Binary logistic regression was used to evaluate the influence of various factors on the two groups (experimental and control). A *p*-value of <0.05 was considered statistical significance.

## 3. Results

Overall, 323 patients with T1DM were approached in the Endocrinology Unit, 194 were eligible, and 140 eligible patients agreed to participate. The 140 patients were randomly allocated to receive the education program or receive routine education (*n* = 70, respectively). In the control group, eight patients revoked their consent due to different job and family difficulties. One patient died in the intervention group due to accidental death. One hundred and thirty-one patients were included in this study; 69 patients were enrolled into the intervention group and 62 were enrolled in the control group.

### 3.1. Participant Characteristics

The baseline data for all 131 patients indicated that the mean age was 36.71 (*S.D*. 12.07) years, 52.7% were women, and 59.54% were married. More than half of the sample were active laborally (58.02%) and 43.51% had a junior high education. The demographic data of the sample are presented in Table 2. No significant differences among demographic characteristic, metabolic, and psychosocial aspect data were found between the intervention and control groups at baseline, thus confirming the homogeneity of the sample.

Table 3 shows the data obtained after the descriptive analysis of the clinical characteristics of the patients who participated in each group at the three points (baseline, 1, and 3 months). Statistical significance was analyzed between groups throughout the evolution of the education program. Significant differences were observed in the target range (*p* = 0.027), the average glucose (*p* = 0.009), with an inverse relationship, the average glucose being lower after the educational program. Significant also were HbA1c (*p* = 0.015) and the sensor use (*p* = 0.036), as well as anxiety (*p* = 0.026) and depression (*p* = 0.004) measured with the Goldberg Scale.

### 3.2. Study Outcomes

A bivariate analysis was performed to analyze the statistical significance between anxiety, depression, diabetes self-care, knowledge, and glycemic control. Anxiety and depression were negatively associated with average glucose and HbA1c level (*p* < 0.05); knowledge and diabetes self-care were positively associated with average glucose and HbA1c level (*p* < 0.05). Diabetes self-care was positively associated with knowledge and negatively with anxiety (*p* < 0.001). Anxiety was also negatively related to the knowledge and high and low ranges (*p* < 0.05) (Table 4).

### 3.3. Binary Logistic Regression

Binary logistic regression was used to evaluate target range, high range, low range, average glucose, HbA1c level, sensor usage, knowledge depression, anxiety, and diabetes self-care three months after the educational program. The two groups were taken as the dependent variable (assignment: 0 = control group, 1 = intervention group), and the assignment of the independent variable are shown in Table 5.

The differences between intervention the group and control group in sensor usage (*Exp* (*B*): 1.048; 95% *CI*: 1.012, 1.84; *p* = 0.008), knowledge (*Exp* (*B*): 0.438; 95% *CI*: 0.336, 0.569; *p* < 0.001), depression (*Exp* (*B*): 1.901; 95% *CI*: 1.483, 2.435; *p* < 0.001), and diabetes self-care (*Exp* (*B*): 1.042; 95% *CI*: 1.003, 1.082; *p* = 0.034) three months after the educational program were significant.

## 4. Discussion

This study evaluated both the glycemic control and the emotional state of the diabetic patient after participating in a structured self-care education program. Whitworth et al. (2016) concluded in their study that lifetime depression anxiety increases the risk of more severe psychological symptoms, hyperglycemia, and difficulties with health behavior in diabetes [33]. Early screening for these disorders may be warranted to maximize health outcomes. Therefore, it was decided in this study to construct a screening with a scale that evaluates both anxiety and depression and is simple, brief, and easy to complete by patients [34].

A previous study conducted in adolescents with T1DM used the Chinese version of the Diabetes Distress Scale, divided into four subscales: emotional burden, physician-related distress, regimen-related distress, and diabetes-related interpersonal distress. Factors associated with higher emotional burden included less communication of diabetes self-management and higher perceived stress levels. However, the authors of this study did not find significant associations between diabetes care activities of diabetes self-management, general self-efficacy, and any domain of diabetes distress. Diabetes care activities were not reported to be associated with higher levels of any domain of diabetes distress [11]. In our study, we found a statistically significant association between diabetes self-care activities and anxiety and depression with an inverse relationship. Better scores in “diabetes self-care activities measures” were associated with lower scores on the Goldberg Scale of anxiety and depression. The data are hardly comparable due to the study population, since Luo and collaborators [11] studied an adolescent population and we studied an adult population over 18 years of age and also different evaluation scales. A previous study concluded that diabetes-related self-care activities reduce perceived stress in people with diabetes, although no significant relationship was found regarding anxiety and depression [35]. The authors of this study observed an increase in knowledge about diabetes management, an improvement in self-care, and greater self-confidence in the patients, which could have influenced their mood and decreased depressive and anxious symptoms.

Diabetes self-care activity measures also showed statistical significance with the knowledge test. Those data contrast with a multicenter, randomized controlled trial conducted to evaluate the effectiveness of a self-efficacy-focused structured education program on adults with type 2 diabetes [36]. We share with these authors the adult population but not the type of diabetes. There are a greater number of education programs in type 2 than T1DM. Jiang and collaborators observed that diabetes distress decreased in each group (intervention and control), but there was no significant difference between the two groups at the 6-month follow-up. In our study, depressive and anxious symptoms decreased significantly at the 3-month follow-up.

Another quasi-experimental trial study found that patients in the intervention group had higher blood sugar compliance rates than the control group [5]. Significant differences between the two groups were found in our study. Improvements in glycemic control demonstrated with the continuous glucose monitoring sensor tool, whose parameters were analyzed at baseline, one and three months after the educational program. Significant differences were observed between the intervention and control group in the target range, average glucose and HbA1c levels.

Diabetes distress had been found to be the strongest independent predictor of metabolic control (measured by HbA1c) [37]. In this study, differences were found between the intervention group and the control group three months after the end of the education program in the level of knowledge, self-care diabetes, and depressive symptoms, and no statistically significant differences were found in anxiety. This indicates that knowledge of diabetes management, diabetes self-care, and depressive symptoms improved after the intensive educational intervention conducted by a nurse educator. The improvement in knowledge is consistent with previous studies evaluating the outcomes of educational interventions in patients with type 1 and 2 diabetes [5,36,38]. Diabetes self-care also improved in some previous clinical trials [36,39]. Improvement of depressive symptoms was a novel finding of this study in the adult population with T1DM. There was a previous study that indicated a higher prevalence of psychological burden as depressive symptoms but in the adolescent population with T1DM.

The current study has several limitations. First, the use of self-report, structured, and closed-ended questionnaires might contain biased responses since closed-ended questions may restrain a patient from expressing everything he or she thinks. For this reason, the authors of this study decided to make a preliminary qualitative study of participants in the educational program [40]. Second, the results’ impact and generalization may be limited because subjects were selected from only one diabetes center. This is a first study, and the authors have the idea of launching a project at the national level to implement the educational program in other centers of the country. Third, although the study population was adult patients, the age range is wide (18–57 years) so the sample may be heterogeneous in this respect. However, the age variable was not statistically significant when compared with the different clinical variables of the study.

## 5. Conclusions

The intensive education program structured in four sessions on insulin administration, blood glucose management, nutrition, and physical exercise with a patient-centered motivational methodology demonstrated effectiveness on patients’ diabetes knowledge, emotional regulation, and self-care. The results demonstrated that the program could help T1DM patients to improve control rates for blood glucose. The continuous glucose monitoring sensor allowed knowing which parameters improved one and three months after the intervention. The hypothesis of the influence of the emotional state on glucose levels was confirmed, mainly influencing the parameters average glucose and glycosylated hemoglobin. The intensive methodology in a few sessions on consecutive days favors patient follow-up, reinforcement of education, and avoids dropouts from the program. Program planning in small groups of four patients favors learning, interpersonal relationships, and support among patients. This study provides a reference management mode for patients with diabetes.

## Figures and Tables

**Table 1:** Structured education program.

Session Theme	Sessions Contents	Learning Methodology
First session. Insulin administration and blood glucose self-analysis.	Insulin device preparation recommendations, maintenance, and conservation of medication Insulin administration zones and rotation and correct technique Capillary glucose self-test review with glucometer Review and recommendations on using the continuous glucose monitoring sensor. Programming of hypoglycemia and hyperglycemia alarms and trend arrows review	Nursing education and simulation Simulated demonstration by patients Resolution of doubts about glucose controls, use of the sensor, and medication administration General review and sharing of main points
Second session. Management of hypoglycemia and hyperglycemia.	Metabolic control goals in adults What is hyperglycemia and how to act What is hypoglycemia and how to resolve Identify possible error or causes that motivate hypoglycemia or hyperglycemia	Nurse educator teaching metabolic control goals Practical exercise for patients. Case studies to comment what actions should be taken depending on glucose levels Resolution of doubts and questions General review and sharing of main points
Third session. Healthy diet adapted to the diabetic patient.	Reading food labels on products Recognition of carbohydrates in food Calculation of carbohydrates by ration each meal Plan a healthy diet example for a full day Calculate carbohydrate rations for each meal	Teaching healthy diet, proportions, and carbohydrate counting Activity: preparation of a one-day diet and carbohydrate counting Resolution of doubts and questions General review and sharing of main points
Fourth session. Physical exercise.	Physiological process that exercise causes in the body Types of exercises recommended Precautions and recommendations before starting exercise Insulin adaptations and previous blood glucose	Exercise instructions, precautions, types of exercise, and intensity Practical scenarios for patients Resolution of doubts and questions General review and sharing of main points

**Table 2:** Sociodemographic variables of patients (*n* = 131).

Variables		
Age (years)	Range	Mean (SD)
	18–57	36.71 (12.07)
Gender	Number	Percentage
Female	69	52.7
Male	62	47.3
Education level		
Elementary	49	37.41
Junior high	57	43.51
Junior college	9	6.87
University or above	16	12.21
Marital status		
Single	36	27.48
Married	78	59.54
Divorced or widowed	17	12.98
Current employment		
No	55	41.98
Yes	76	58.02

**Table 3:** Analysis of baseline, 1 month, and 3 months evaluations of different groups.

	Experimental Group (*n* = 69)			Control Group (*n* = 62)			Spearman’s Rho	*p*-Value
Variables	Baseline *M* +/− SD	1 Month *M* +/− SD	3 Months *M* +/− SD	Baseline *M* +/− SD	1 Month *M* +/− SD	3 Months *M* +/− SD		
*Sensor measurements* Target range ^1^	54.805 (18.205)	65.410 (19.345)	62.487 (19.744)	54.851 (18.246)	54.238 (18.098)	53.824 (17.218)	0.168	0.027 *
High range ^2^	41.829 (18.942)	31.769 (19.679)	34.681 (20.008)	41.693 (19.127)	41.846 (18.976)	41.785 (18.875)	−0.138	0.070
Low range ^3^	3.512 (3.099)	2.820 (2.624)	2.931 (2.724)	3.456 (3.078)	3.916 (3.229)	4.391 (4.126)	−0.036	0.637
Average glucose	190.024 (41.763)	172.103 (46.703)	176.369 (44.79)	189.140 (40.825)	188.458 (40.734)	189.678 (41.287)	−0.199	0.009 *
HbA1C level	7.778 (0.996)	7.374 (1.019)	7.481 (1.026)	7.794 (0.987)	7.806 (1.018)	7.788 (0.948)	−0.184	0.015 *
Sensor usage (%)	80.871 (17.601)	87.769 (12.854)	87.989 (13.528)	81.745 (18.029)	82.016 (17.549)	81.512 (18.348)	0.159	0.036 *
*Knowledge test*	7.39 (1.32)	8.077 (1.036)	8.087 (1.219)	7.28 (1.19)	7.32 (1.275)	7.19 (1.452)	0.106	0.151
*Goldberg Scale* Depression	2.781 (2.067)	2.128 (1.341)	2.017 (1.611)	2.812 (1.758)	2.925 (2.025)	2.937 (2.048)	−0.216	0.004 *
*Goldberg Scale* Anxiety	4.366 (2.447)	2.923 (1.612)	2.894 (1.018)	4.248 (2.126)	4.358 (2.251)	4.342 (2.238)	−0.166	0.026 *
*Diabetes Self-Care Activities Measure*	31.268 (9.268)	33.077 (8.174)	33.235 (8.254)	31.552 (10.126)	30.947 (9.147)	30.898 (8.474)	0.028	0.705

^1^ % time in target range (70–180 mg/dl); ^2^ % time in high range (>180 mg/dL); ^3^ % time in low range (<70 mg/dl); * Indicates significant (*p* < 0.05).

**Table 4:** Bivariate analysis.

Variables	*Goldberg Scale Anxiety* Spearman’s Rho (*p*-Value)	*Goldberg Scale Depression* Spearman’s Rho (*p*-Value)	*Diabetes Self-Care Activities Measure* Spearman’s Rho (*p*-Value)	*Knowledge Test* Spearman’s Rho (*p*-Value)
Age	0.064 (0.475)	0.046 (0.606)	−0.167 (0.056)	−0.101 (0.249)
Target range	0.110 (0.158)	0.030 (0.700)	0.023 (0.771)	−0.190 (0.012) *
High range	−0.167 (0.030) *	−0.073 (0.345)	0.073 (0.347)	0.269 (0.000) *
Low range	−0.159 (0.040) *	0.036 (0.641)	−0.189 (0.014) *	0.100 (0.189)
Average glucose	−0.197 (0.010) *	−0.167 (0.030) *	0.239 (0.002) *	0.255 (0.001) *
HbA1c level	−0.200 (0.009) *	−0.185 (0.017) *	0.252 (0.001) *	0.266 (0.000) *
Sensor usage (%)	−0.068 (0.383)	0.116 (0.134)	−0.127 (0.100)	0.059 (0.442)
Knowledge test	−0.629 (0.000) *	−0.375 (0.000)	0.266 (0.000) *	
Goldberg Scale—Depression	0.467 (0.000) *			
Goldberg Scale—Anxiety			−0.472 (0.000) *	
*Diabetes Self-Care Activities Measure*	−0.472 (0.000) *	−0.505 (0.000) *		

* Indicates significant (*p* < 0.05).

**Table 5:** Factors of two groups through a Binary Logistic regression.

Variables	B	S.E.	Wals	*p*	Exp (B)	EXP(B) 95% C.I.
Gender	−0.082	0.280	0.087	0.768	0.921	0.532, 1.593
Age	−0.004	0.012	0.128	0.721	0.996	0.972, 1.020
Target range	0.101	0.071	1.979	0.159	1.106	0.961, 1.272
High range	0.114	0.073	2.455	0.117	1.120	0.972, 1.292
Low range	0.155	0.081	3.680	0.055	1.168	0.997, 1.368
Average glucose	−0.057	0.033	2.911	0.088	0.945	0.886, 1.008
HbA1c level	1.996	1.461	1.868	0.172	7.362	0.420, 128.930
Sensor usage (%)	0.047	0.018	7.053	0.008 *	1.048	1.012, 1.084
Knowledge	0.827	0.134	37.955	0.000 *	0.438	0.336, 0.569
Depression	0.642	0.126	25.768	0.000 *	1.901	1.483, 2.435
Anxiety	−0.135	0.091	2.207	0.137	0.874	0.731, 1.044
Diabetes Self-Care Activities Measure	0.41	0.019	4.483	0.034 *	1.042	1.003, 1.082

* Indicates significant (*p* < 0.05).

## Data Availability

Due to patient data protection and confidentiality agreement with the Ethics Committee, there is no published database. However, if any reader is interested, please contact the correspondence author and principal investigator of this study, Rocío Romero Castillo (rocio.romero@cruzroja.es).

## Supplementary Materials

The following supporting information can be downloaded at: https://www.mdpi.com/article/10.3390/ijerph192316364/s1, Table S1: CONSORT checklist.
